# Magnetic Property, Heat Capacity and Crystal Structure of Mononuclear Compounds Based on Substitute Tetrazole Ligand

**DOI:** 10.3390/molecules28186633

**Published:** 2023-09-15

**Authors:** Hui Zheng, Jipeng Luo, Xiaoqin Wang, Nan Yin, Beibei Zhang, Xuezhen Gao, Zongzheng Zhang, Quan Shi, Junshen Liu

**Affiliations:** 1 School of Chemistry and Materials Science, Ludong University, Yantai 264025, China; wang20212023@163.com (X.W.); zhbb0302@163.com (B.Z.); gaoxuez@163.com (X.G.); zhangzongzheng@m.ldu.edu.cn (Z.Z.); 2 Thermochemistry Laboratory, Dalian Technology Innovation Center for Energy Materials Thermodynamics, Liaoning Province Key Laboratory of Thermochemistry for Energy and Materials, Dalian National Laboratory for Clean Energy, Dalian Institute of Chemical Physics, Chinese Academy of Sciences, Dalian 116023, China; luojp@dicp.ac.cn (J.L.); yin7310@dicp.ac.cn (N.Y.); shiquan@dicp.ac.cn (Q.S.)

**Keywords:** mononuclear compounds, magnetic property, heat capacity, Schottky anomaly, crystal structure

## Abstract

Three mononuclear compounds formulated as {M[(2-1H-tetrazol-5-yl)pyridine]_2_(H_2_O)_2_} (M = Fe^II^ (**1**), Co^II^ (**2**), Cu^II^ (**3**)) were reported and synthesized. Their space group is monoclinic, *P*2_1_/c, revealed by single-crystal X-ray diffraction. Antiferromagnetic interactions exist in Compounds **1**, **2** and **3**, as evidenced by magnetic and low-temperature heat capacity measurements. In addition, their thermodynamic functions were determined by a relaxation calorimeter, indicating that Compound **1** exhibits a Schottky anomaly in low-temperature heat capacity. This work can provide an important fundamental basis for the research of the thermophysical properties of molecular-based magnetic materials.

## 1. Introduction

Single-molecule magnets (SMMs) are the real molecular magnets of nano size (with molecular diameter between 1 and 2 nm). They are the first discrete and non-interacting nano-sized molecular units in a magnetic sense as opposed to magnets composed of three-dimensional extended lattices (such as metal, metal oxide, metal complex, etc.). In recent years, due to the great application potential of SMMs in quantum computing, high-density information storage, etc., more and more scientific and technological personnel and teams have devoted a great amount of research resources to it. The following are the characteristics of SMMs: presence of a very slow magnetic relaxation phenomenon at a low temperature (blocking temperature), exhibition of superparamagnetic properties, the appearance of a magnetization quantum tunneling effect and a hysteresis loop during low-temperature direct current field magnetization [1,2,3,4]. Since the 12-manganese core cluster compound [Mn_12_O_12_(O_2_CMe)(H_2_O)_4_] [2] was found to have the properties of a single-molecule magnet, a large number of complexes with similar properties have been successfully synthesized. Now, there have been five major single-molecule complex systems, including Fe-based, Co-based, Mn-based, V-based, and Ni-based. An effective strategy for designing and synthesizing SMMs is to connect paramagnetic centers through short bridges while selecting different auxiliary organic co-ligands to adjust the structure and coordinate polymer dimensions. Nitrogen-rich tetrazolium ligands have been found to exhibit versatile coordinates on patterns and a tendency to build rigid coordination bond networks. In particular, tetrazolium and its metal compounds can be used as multidentate ligands or bridging ligands to construct coordination polymers [5,6,7,8].

Tetrazolium ligands, containing four nitrogen atoms, can provide multiple coordination sites and hydrogen bonding acceptors, making them highly suitable for coordination and supramolecular chemistry. The cycloaddition of 1-H-tetrazole has further accelerated the development of tetrazole-like ligands that exhibit strong metal binding through bidentate chelation, thus highlighting the potential of substituted tetrazole ligands [9,10]. In particular, 5-substituted tetrazoles can be obtained through environmentally friendly methods, such as those of Sharpless [11], Xiong [12] and their coworkers, who reported some simple approaches to synthesize these substituted tetrazole ligands. Furthermore, the combination of tetrazole with metal allows for unusual extensions of structures due to the way tetrazole binds to metals and the presence of a hydrogen-bonding network in the resulting compounds based on the tetrazole ligands.

In this work, we designed three mononuclear compounds containing transition metal ions and tetrazole derivatives, with the following formula: {M^II^[(2-1H-tetrazol-5-yl)pyridine]_2_(H_2_O)_2_}(M^II^ = Fe (**1**), Co (**2**) and Cu(**3**)). The crystal structure, magnetism and heat capacity (1.9–300 K) of these three compounds were comprehensively studied. At the same time, their heat capacity values and related thermodynamic functions at smooth temperatures were obtained by fitting the experimental data. More importantly, these complexes exhibited *Schottky* anomalies in the low-temperature heat capacity curves, validating the antiferromagnetic exchange interactions determined by magnetic measurements.

## 2. Results and Discussion

### 2.1. Crystal Structure

**Figure 1:**
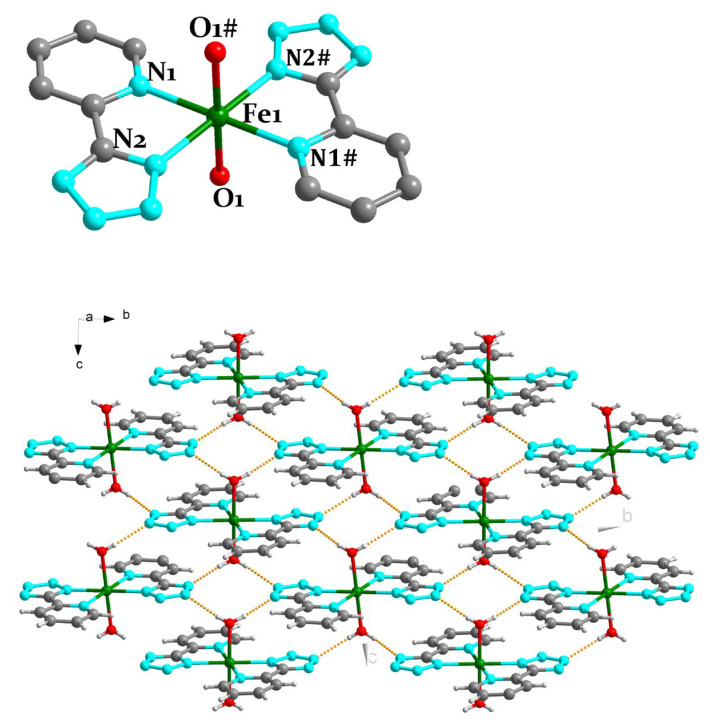
(**top**) Crystal structure of Compound **1**. (**bottom**) H-bonds in Compound **1** along *b* axis. For clarity, most hydrogen atoms are omitted. Green, gray, bluish violet, and red spheres represent Fe^II^, C, N, and O atoms, respectively. #, symmetry transformations used to generate equivalent atoms: −*x* + 2, −*y* + 1, −*z*.

Single-crystal X-ray diffraction shows that these compounds all crystallize in the monoclinic space group *P*2_1_/c. Due to the structural similarity of Compounds **1**–**3**, the Fe^II^ analogue is chosen as an example to describe their detailed structures (Figure 1 (top)). The crystallographic data are presented in Table 1. The centrosymmetric crystal unit contains a Fe{2-(1H-tetrazol-5-yl)pyridine}_2_(H_2_O)_2_ mononuclear unit. The central Fe^II^ ion, located in an octahedral environment, is coordinated with four planar nitrogen atoms from two ligands and two axial oxygen atoms from two coordinated water molecules. Thus, the Fe^II^(1) ion adopts a FeN_4_O_2_ octahedral geometry. The three bond lengths of Fe^II^(1)-N are in the range of (2.1537 ± 0.0002)–(2.2007 ± 0.0002) Å, and the two bond lengths of Fe^II^(1)-O are (2.1458 ± 0.0002) Å, respectively, indicating that the Fe^II^ ion is in the high spin state [13,14,15]. The N(1)-Fe^II^(1)-O(1) and N(1)-Fe^II^(1)-O(2) angles are equal to (90.048 ± 0.002)° (Table 2). In the crystal structure of Compound **1**, the bond lengths of the adjacent units (O-H∙∙∙N) are 2.757–2.831 Å (Figure 1 (bottom)), so the adjacent structural units are connected into a two-dimensional layer by hydrogen bonds (Table 3).

**Figure 2:**
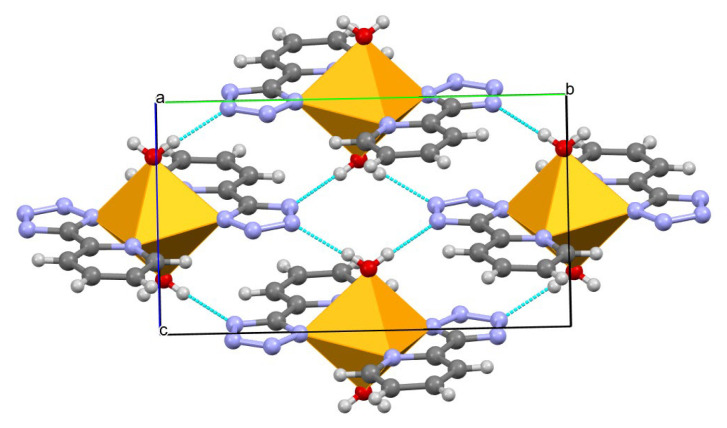
H-bonds in Compounds **2** and **3** along *b* aixs. For clarity, most hydrogen atoms are omitted. Co^II^/Cu^II^, orange; C, gray; N, light blue; O, red.

For Compound **2**, the central Co^II^ ion adopts CoN_4_O_2_ octahedral geometry. The bond lengths of Co^II^-N and Co^II^-O are in the range of (2.1142 ± 0.0002)–(2.1461 ± 0.0002) and (2.1257 ± 0.0002) Å, indicating that the Co^II^ is in the high-spin state [16]. As shown in Table 2, the N(1)-Co^II^(1)-O(1) and N(1)-Co^II^(1)-O(2) angles are equal to 90°. In Compound **3**, the bond lengths of Cu^II^-N and Cu^II^-O are in the range of (2.0144 ± 0.0002)–(2.0415 ± 0.0002) and (2.4205 ± 0.0002) Å, respectively. It is worth noting that the N-Cu^II^(1)-O angles are deviated by 90° (87.45°–92.55°); thus, the central Cu^II^ ion is located in a distorted octahedron, maybe due to the Ginger Taylor effect of the Cu^II^ ion. Compared with Compound **1**, the hydrogen bonds in Compounds **2** (2.013–2.057 Å) and **3** (2.064–2.161 Å) are much shorter, also linking the adjacent building blocks into a 2D lamellar structure (Figure 2).

Therefore, there are indeed strong O-H···N hydrogen bonds building a two-dimensional layer parallel to the (1 0 0) plane, but the weak C-H···O bond links these layers, building a three-dimensional network for all the three compounds.

### 2.2. Powder X-ray Diffraction (XRD) 

**Figure 3:**
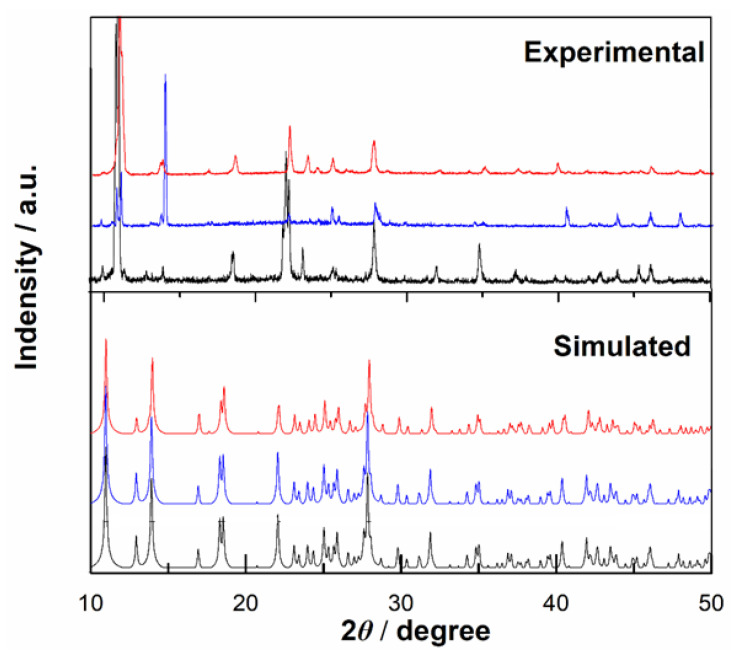
Powder X-ray diffraction patterns of experiment and simulated ones for Compounds **1**–**3**. Color identification: red, Compound **1**; blue, Compound **2** and black, Compound **3**, respectively.

Phase purity was determined by powder X-ray diffraction (XRD) for large volumes of samples. The experimental XRD spectra are consistent with the simulated spectra collected from single-crystal X-ray diffraction data, indicating that the bulk sample and the measured single crystal structure are completely identical. As shown in Figure 3, compared to the simulated spectrogram, some peaks shift and weaken due to the uniformity and anisotropy of the sample, as well as differences in the test sample preparation.

### 2.3. Fourier Transform Infrared (FTIR) Characterization 

**Figure 4:**
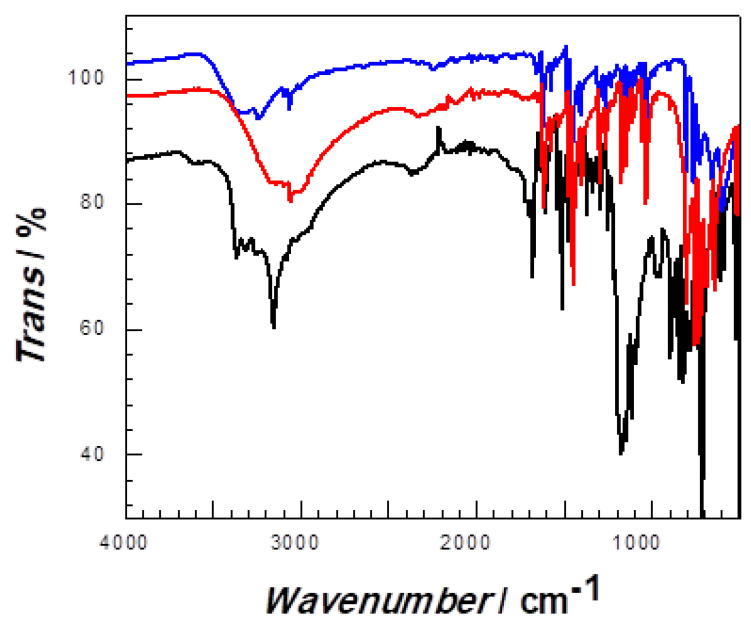
Infrared spectra of Compounds **1**, **2** and **3**. Color identification: red, Compound **1**; blue, Compound **2** and black, Compound **3**, respectively.

The infrared characterization of Compounds **1**–**3** is shown in Figure 4, from which it can be seen that the peak at 823 cm^−1^ is caused by the out-of-plane bending vibration of C-H on para-disubstituted rings, and the stretching vibration of the C-C quinonoid ring appears at 1128 cm^−1^. The peaks at 1487 cm^−1^ are designated to the stretching vibration of the C-C benzenoid ring; the peaks at 3053, 3218, 3230 cm^−1^ in compounds are attributed to the O-H telescopic vibration of the coordination water molecules in the compounds; and the peaks at 1608, 1609, 1615 cm^−1^ are assigned to the C=N peaks of the pyridine and tetrazolium rings. As reported in the literaure [17], the peaks observed at 446–417 cm^−1^ contributed to the bonds of M^II^-N. (M = Fe^II^ Co^II^ or Cu^II^ for Compounds **1**–**3**).

### 2.4. Magnetic Properties 

**Figure 5:**
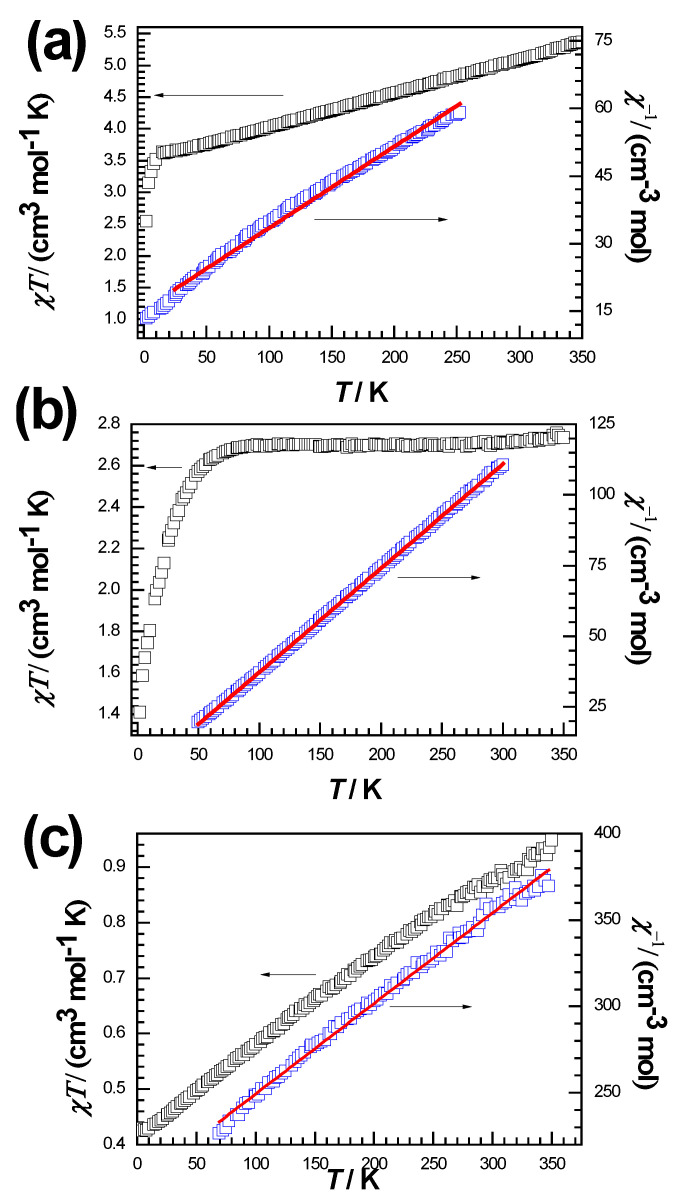
Temperature-dependent χ_M_T and χ_M_^−1^ of Compounds **1** (**a**), **2** (**b**) and **3** (**c**) under 1000 Oe. The red lines represent the fit to the magnetic data.

To confirm the magnetic behavior of Compounds **1**–**3**, magnetic susceptibility measurements were carried out under an applied electric field of 1000 Oe in the temperature range of 2–350 K (Table S2). For Compound **1**, the χ_M_T value around 300 K is 5.10 cm^3^ K mol^−1^ (Figure 5a), much larger than the expected magnetic isolation spin value of only 3.0 cm^3^ K mol^−1^ (S = 2, g = 2.0). The value of χ_M_T decreases almost linearly to 3.63 cm^3^ K mol^−1^ at 8 K with decreasing temperature. When the temperature is reduced further, the minimum magnetization appears at 2 K. This magnetic behavior indicates the existence of antiferromagnetic coupling. The Weiss temperature obtained by Curie Weiss fitting in the temperature range of 50–300 K is −85.67 K, and the calculated Curie constant is 5.55 cm^3^ K mol^−1^, which is consistent with the abovementioned antiferromagnetic intermolecular interaction of Compound **1**.

Compound **2** is a Co^II^ (3d^7^) compound with unpaired electrons, which corresponds to the magnetic moment of Co^II^ (Figure 5b). The χ_M_T value of 2.70 cm^3^ K mol^−1^ at 300 K is larger than the expected pure value of the magnetic isolation spin of 1.87 cm^3^ K mol^−1^ (for Co^II^, S = 3/2, g = 2.0), indicating that there is an unquenched orbital moment that contributes to the magnetic susceptibility [18]. The value of χ_M_T remains almost constant above 80 K, and then decreases to a minimum of 1.41 cm^3^ K mol^−1^ as the temperature decreases to 2 K. This fact indicates the presence of antiferromagnetic coupling in Compound **2**. The magnetic susceptibility data at 50–300 K conform to the Curie–Weiss law, and the calculated Curie constant of 2.78 cm^3^ K mol^−1^ is near to the experimental χ_M_T value. The obtained Weiss temperature is −1.27 K, and this negative value indicates the presence of predominant antiferromagnetic coupling [19,20,21].

For Compound **3** (Figure 5c), the value of χ_M_T at 300 K is 0.870 cm^3^ K mol^−1^, which is in agreement with the expectation of 0.75 cm^3^ K mol^−1^ for one Cu^II^ ion (S = 1/2, g = 2.0). As Compound **3** is cooled, the χ_M_T value decreases almost linearly, reaching a minimum of 0.428 cm^3^ K mol^−1^ at about 2 K. This behavior suggests that Compound **3** exhibits antiferromagnetic coupling, which could explain the observed drop in χ_M_T values at room temperature. The χ_M_T value at low temperature corresponds to the expected value (0.375 cm^3^ K mol^−1^) for the S = 1/2 ground state with a g value of 2.0.

The magnetic exchange between metal ions is achieved through hydrogen bonds formed between oxygen and nitrogen; due to the weak interaction of hydrogen bonds, the magnetic interaction between copper ions is relatively weak. The difference in behavior among the three complexes (Figure 5) can be qualitatively ascribed to the presence of magnetic anisotropy (zero-field splitting, ZFS, of S = 3/2 leading to ms = ±2, ±3/2 and ±1/2 sublevels with different energy) for **1** and an antiferromagnetic exchange coupling between the two Co(II) ions in **2**.

**Figure 6:**
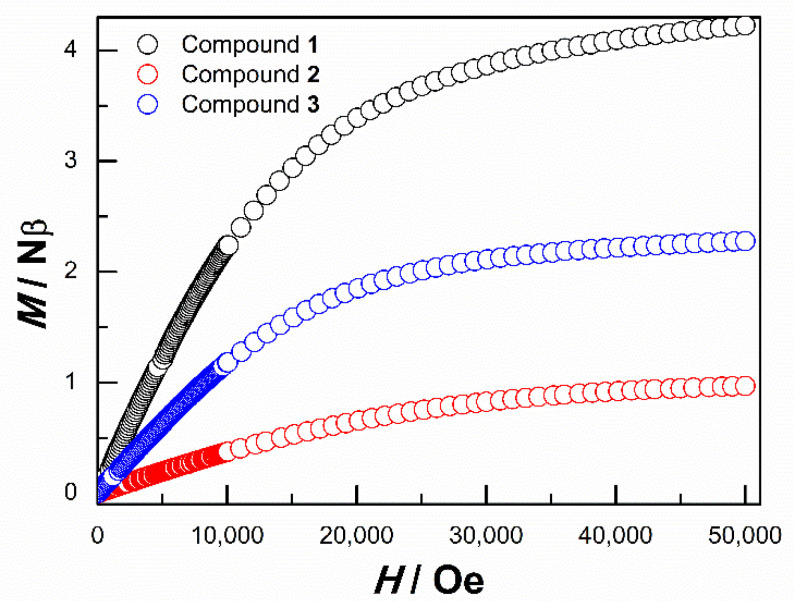
Isothermal field-dependent magnetization of Compounds **1**–**3** at 2 K.

Further confirmation of the ground-state spin at low temperatures comes from the isothermal magnetization measurements at 2 K (Figure 6). It can be found that the magnetization (M) of Compounds **1**–**3** varies quasi-linearly with the applied field (H) up to 50,000 Oe. The magnetization at 50,000 Oe is 4.23 μB/Fe^2+^ and 2.27 μB/Co^2+^, respectively. In particular, the magnetization at 50,000 Oe is only 0.95 μ_B_/Cu^2+^, which is much less than the spin saturation value of 1.73 μ_B_/Cu^2+^ for Cu^2+^ with S = 1/2. This phenomenon also demonstrates the presence of strong antiferromagnetic coupling in Compound **3**.

### 2.5. Heat Capacity and Thermodynamic Functions 

**Figure 7:**
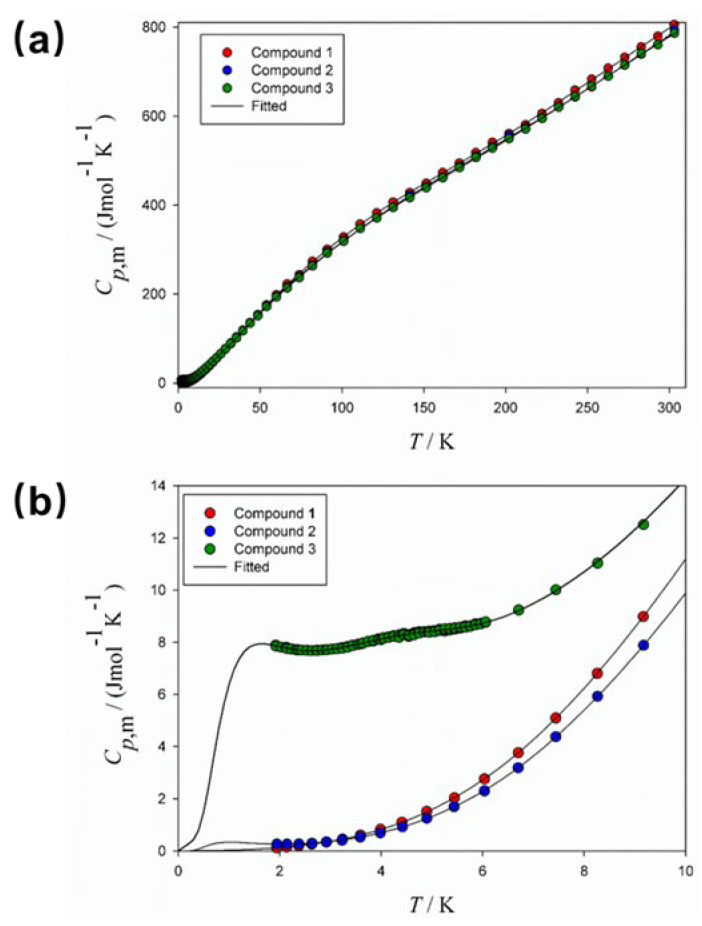
Experimental heat capacity curves of Compounds **1**–**3** at 1.9–300 K (**a**) and below 10 K (**b**).

Heat capacity is a fundamental thermophysical property of materials, and precise calorimetric measurement can be used as a potent characterization tool to obtain microscopic and macroscopic information of substances. Furthermore, phase transitions discovered using heat capacity measurements are direct and obvious probes to elucidate changes in the condensed state of matter [22]. Therefore, to further investigate the properties of these compounds, their low-temperature heat capacities were determined by a relaxation calorimeter. Experimental heat capacity data (*C_p_*_,m_) for Compounds **1**–**3** are listed in Table S3 and plotted in Figure 7. The heat capacity rises steadily as the temperature rises, and above 10 K, there are no thermal anomalies. Interestingly, *C_p_*_,m_ peaks appear below 10 K, with the most pronounced peak for Compound **3** (Figure 7b). The hump in *C_p_*_,m_ is usually due to the *Schottky* anomaly [23,24,25].

Overall heat capacity of a substance can be viewed as the sum of the energy contributions of magnetism, electrons, lattice vibrations, and other features. Usually, the lattice heat capacity is the dominant contributor. At lower temperatures (<10 K), the lattice heat capacity decreases rapidly, and the other contributors can then be determined through fitting the experimental data to an appropriate theoretical model. For Compounds **1**–**3**, the following theoretical equations were adopted to fit the heat capacity data at 1.9–9 K:

*C_p,m_ =* γ*T* + B_3_*T*^3^ + B_5_*T*^5^ + B_7_*T*^7^ + *C*_sch_ + *C*_asw_, (1)

*C*_sch_ = *n*_sch_*R*(θ/*T*)^2^exp(θ/*T*)/(1 + exp(θ/*T*))^2^ (2)

*C*_asw_ = B_asw_*T*^3^exp(−Δ/*T*), (3)

where the linear term γ*T* represents the lattice defects or vacancies of the sample; the odd power of temperature stands for the lattice heat capacity contribution; *C*_sch_ is used to fit the *Schottky* heat capacity, and an antiferromagnetic heat capacity contribution *C*_asw_ is included in the model [26]. A simple two-level model is adopted to fit the *Schottky* heat capacity as shown in Equation (2), where *R* is the ideal gas constant. It is well known that the *Schottky* anomaly is present in systems including hyperfine splitting, zero-field splitting, tunnel splitting, *Zeeman* splitting, and so on. For these three coordination compounds, the *Schottky* heat capacity contribution is expected and found, but further investigation is required to ascertain its cause. The corresponding fitted parameters for Compounds **1**–**3** can be found in Table S4. Note that since the antiferromagnetic heat capacity *C*_asw_ in Equation (3) has a similar *T*^3^ term to that of the lattice heat capacity, they cannot be clearly distinguished. Therefore, the low-temperature fitting parameters, particularly those for the lattice and antiferromagnetic heat capacity, have relatively limited physical significance, but the thermodynamic data calculated from these fittings are valid. In addition, Compound **3** has a much higher γ value (1.3445 J·mol^−1^·K^−2^) than Compounds **1** and **2**, which could be explained by the significant electronic contribution of Cu^2+^ at low temperatures, leading to the apparent *Schottky* anomaly [27].

Above 50 K, a combination of *Debye* and *Einstein* functions was used to fit the heat capacities of Compounds **1**–**3**:

*C*_*p*,*m*_ = *n_D_D*(*Θ*_*D*_) + *n*_*E*,1_*E*(*Θ*_*E*,1_) + *n*_*E*,2_*E*(*Θ*_*E*,2_), (4)

where *D*(*Θ_D_*), *E*(*Θ_E,1_*), and *E*(*Θ_E,2_*) are *Debye*, low-temperature *Einstein*, and high-temperature *Einstein* function, respectively; n*_D_*, *Θ_D_*, n*_E,1_*, *Θ_E,1_*, n*_E,2_*, and *Θ_E,2_* are fitted parameters (Table S4), and the sum of n*_D_*, n*_E,1_* and n*_E,2_* should be approximately the number of atoms in the compound.

In order to smoothly connect the fitted curves for the low- and high-temperature regions, the heat capacity values at 5–75 K were fitted to an arbitrary polynomial equation with no physical meaning:

*C_p_*_,m_ = A_0_*T*^0^ + A_1_*T*^1^ + A_2_*T*^2^ + A_3_*T*^3^ + A_4_*T*^4^ + A_5_*T*^5^ + A_6_*T*^6^+ A_7_*T*^7^. (5)

**Figure 8:**
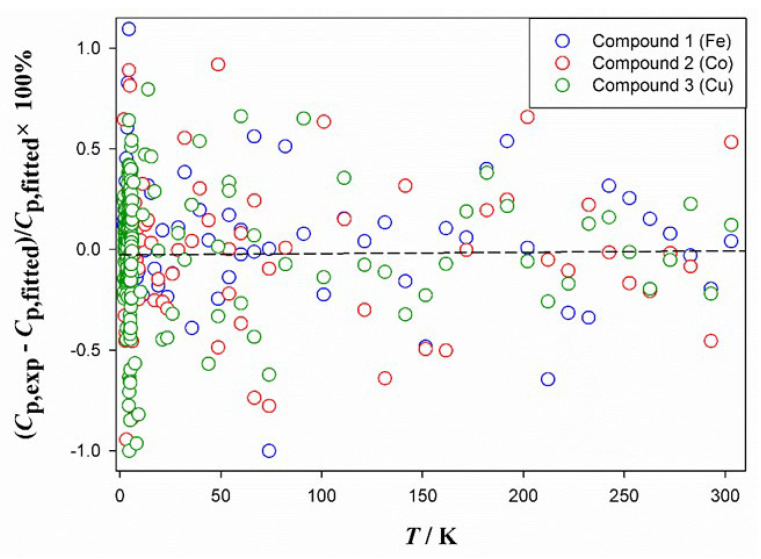
Plot of percentage deviations of experimental heat capacities against temperature from the fitted values.

All fitted parameters, root mean square (%RMS) errors, and corresponding temperature ranges are listed in Table S4. As shown in Figure 8, the percentage deviation of the experimental heat capacities from the fitted data is within ±1% and ±0.5% at 1.9–20 K and 20–300 K, respectively. Deviations are randomly distributed and the %RMS errors of all fits are less than 0.53. Therefore, the above fitting Equations (1)–(5) can accurately represent the experimental heat capacity data.

Standard molar heat capacity, entropy, and enthalpy are useful basic thermodynamic parameters. For example, the entropy gain at phase transition is a diagnostic metric for understanding the chaotic changes of the system [22]. Based on the above fitting and basic thermodynamic laws, the standard thermodynamic functions of Compounds **1**–**3** were calculated [26,27], and the results are presented in Table S5. The standard molar heat capacity, entropy and enthalpy at 298.15 K and 0.1 MPa were determined to be $C_{p,\;m}^{o}$ = (793.4 ± 7.9), (776.7 ± 7.8) and (774.0 ± 7.7) J·K^−1^·mol^−1^;${∆S}_{m}^{o}$ = (836.4 ± 8.4), (821.1 ± 8.2) and (838.4 ± 8.4) J·K^−1^·mol^−1^; and ${∆H}_{m}^{\;o}$ = (126.3 ± 1.3), (123.9 ± 1.2) and (123.5 ± 1.2) kJ·mol^−1^ for Compounds **1**, **2** and **3**, respectively.

## 3. Materials and Methods

### 3.1. Materials

All chemicals were purchased commercially at analytical grade and used without any purification (Table S1). Compounds **1**–**3** were prepared by the diffusion method at room temperature, and the solvent system and the ratio of metal to ligand greatly affected the formation of the final crystals. Holding other conditions of the reaction constant, single crystals of the compounds in highest yield suitable for characterization were obtained only when the ratio of metal to ligand was 1:3. The combination and ratio of mixed solvents were also important factors, and crystals with good morphology can only be obtained in a mixed solvent of ethanol and water solvent. Notably, Compounds **1**–**3** are all stable in air.

### 3.2. Synthesis of [Fe{2-(1H-tetrazol-5-yl)pyridine}_2_(H_2_O)_2_] (**1**)

Compound **1** was prepared by the layered diffusion method. A total of 2 mL of the Fe(ClO_4_)_2_·6H_2_O (0.01 mmol, 3.920 mg) aqueous solution was added dropwise to the bottom of the test tube, and then 4 mL of a mixed solvent of methanol–water (volume ratio 1:1) was slowly added as a buffer layer. Subsequently, 2 mL of a methanol solution containing 2-(1H-tetrazol-5-yl)pyridine (0.02 mmol, 4.962 mg) was added dropwise to the uppermost layer of the test tube. Finally, the tube was sealed with a parafilm and left to stand at room temperature for several days to obtain yellow bulk crystals (yield: 40% based on Fe^II^). The following are the C_12_H_12_N_10_O_2_Fe elemental analysis: calculated values (%): C 37.48, H 3.12, N 36.44; experimental values: C 37.42, H 3.15, N 36.46.

### 3.3. Synthesis of [Co{2-(1H-tetrazol-5-yl)pyridine}_2_(H_2_O)_2_] (**2**) and [Cu{2-(1H-tetrazol-5-yl)pyridine}_2_(H_2_O)_2_] (**3**)

Compounds **2** and **3** were synthesized in the same way, except Co(OA_C_)_2_·4H_2_O was replaced by CuCl_2_·2H_2_O to obtain light blue bulk crystals for Compound **3**, taking the synthesis of Compound **2** as an example for illustration. A total of 2 mL of the Co(OA_C_)_2_·4H_2_O (0.01 mmol, 2.490 mg) aqueous solution was added dropwise to one side of the H-type tube; 2 mL of a methanol solution containing 2-(1H-tetrazol-5-yl)pyridine (0.02 mmol, 4.962 mg) was added dropwise to the other side of the H-type tube; subsequently, a mixed solvent of methanol–water (volume ratio 1:1) was slowly added as a buffer layer. Finally, the tube was sealed with a parafilm and left to stand at room temperature for several weeks to obtain orange bulk crystals (yield: 45% based on Co^II^ for Compound **2**, 55% based on Cu^II^ for Compound **3**). The following are the C_12_H_12_N_10_O_2_Co elemental analysis calculated values (%): C 37.18, H 3.10, N 36.15; experimental values: C 37.21, H 3.11, N 36.13. The following are the C_12_H_12_N_10_O_2_Cu elemental analysis: calculated values (%): C 36.75, H 3.06, N 35.73; experimental values: C 36.70, H 3.12, N 35.69.

### 3.4. Physical Characterization

Powder X-ray diffraction (PXRD) was performed on an X-pert Pro-1 automatic powder X-ray diffractometer at room temperature using Cu-*K*_α_ radiation (*λ* = 1.5418 Å) with a scan rate of 6° per minute. The experimental powder X-ray diffraction patterns of Compounds **1**–**3** and their simulated patterns based on the crystal data are shown in Figure 3. Infrared (IR) spectra were recorded with a Tensor 27 spectrometer (Bruker Optics, Ettlingen, Germany) in the range of 4000–400 cm^−1^ using crystalline samples on KBr pellets. 

Elemental analyses (C, H, N) were performed on a Vario EL III fully automated trace element analyzer by high-temperature combustion reaction. Our measurements were performed by dissolving the complex in water to make a dilute solution.

### 3.5. X-ray Structure Determination

All the single-crystal X-ray diffraction experiments were performed on a Bruker Smart Apex CCD diffractometer equipped with graphite monochromatized Mo Kα radiation (λ = 0.71073 Å) using *ω*-*φ* scan modes. Lorentz polarization and absorption corrections were applied. The structure was solved by direct methods using SHELXS-97 and refined by means of full-matrix least squares procedures on *F*^2^ with a SHELXL-97 program [28,29].

All non-H atoms were located using subsequent *Fourier* difference methods and refined anisotropically. In all cases, hydrogen atoms were placed in their calculated positions and thereafter allowed the ride on their parent atoms. Experimental details for the structural determination of these compounds are summarized in Table 1, the selected bond lengths and bond angles data are presented in Table 2, and the hydrogen bonds are listed in Table 3. The cif and checkCIF files are available in Supplementary Materials.

### 3.6. Magnetic and Calorimetric Measurements

A SQUID MPMS XL-7 magnetometer from Quantum Design was used to determine the magnetic susceptibility of Compounds **1**–**3**. Constant field magnetic studies were performed at a rate of ±1 K/min over a temperature range of 2 to 300 K under a field of 1000 Oe. Data were corrected for the diamagnetic contribution calculated from Pascal constants and sample holder [30].

The heat capacity of the samples was determined using a Quantum Design relaxation calorimeter in the temperature range from 1.9 to 300 K. The measurement accuracies for high-purity copper and *α*-Al_2_O_3_ cylinders were estimated to be within ±2% and ±1% at 1.9 to 20 K and 20 to 300 K, respectively [31]. The sample preparation steps and heat capacity measurement procedures for crystals are detailed in the literature, and it has been verified that the accuracy is within ±3% (<20 K) and ±1% (20–300 K), respectively [32,33].

Typically, the sample preparation process involves mixing the sample with the copper strip in a copper cup and compressing it into particles using a stainless steel mold. The copper cup is formed from 0.025 mm thick pure copper foil with a mass fraction purity of 0.99999. The particle diameter is approximately 2.8 mm and the height is 1–3 mm. Detailed information on sample preparation and heat capacity measurement procedures can be found in relevant work [34]. The sample masses in the heat capacity measurement are 7.71 mg, 7.02 mg, and 5.74 mg of the crystalline sample for Compounds **1**, **2** and **3**, respectively.

## 4. Conclusions

In summary, three mononuclear compounds built from 2-(1H-tetrazol-5-yl)pyridine were successfully synthesized and carefully characterized. Their space group of *P*2_1_/c was determined by single-crystal X-ray diffraction. Magnetic measurements indicated the presence of antiferromagnetic interactions in these compounds, which were supported by their low-temperature heat capacity data. In addition, their heat capacity values were fitted by theoretical or empirical functions, and then the standard thermodynamic functions of these compounds were calculated according to the fundamental laws. These results can provide an important fundamental basis for the research of the thermophysical properties of molecular-based magnetic materials.

## Figures and Tables

**Table 1:** Crystal Data and Structure Refinement Parameters for Compounds **1**–**3**.

Compound	1	2	3
Empirical formula	C_12_H_12_N_10_O_2_Fe	C_12_H_12_N_10_O_2_Co	C_12_H_12_N_10_O_2_Cu
CCDC	2,239,647	2,239,654	2,239,654
Formula weight	384.17	387.25	391.86
Crystal system	Monoclinic	Monoclinic	Monoclinic
Space group	*P*2_1_/c	*P*2_1_/c	*P*2_1_/c
Temperature/K	296.15 ± 2	297.0 ± 2	296.15 ± 2
*a* (Å)	8.1032 ± 0.0009	8.0925 ± 0.0018	8.0925 ± 0.0018
*b* (Å)	12.9127 ± 0.0014	12.870 ± 0.003	12.870 ± 0.003
*c* (Å)	7.3470 ± 0.0008	7.3128 ± 0.0017	7.3128 ± 0.0017
*α* (°)	90.00	90.00	90.00
*β* (°)	96.092 ± 0.002	96.070 ± 0.004	96.070 ± 0.004
*γ* (°)	90.00	90.00	90.00
*V*/Å^3^	764.41 ± 0.14	757.3 ± 0.3	757.3 ± 0.3
*Z*	2	2	2
Density calculated/g cm^−3^	1.669	1.698	1.718
*µ*/mm^−1^	1.019	1.166	1.475
*F*(000)	392.0	394.0	398
Independent reflections	4560	7905	10,938
Data/restraint/parameters	1765/0/120	1891/0/139	1949/0/139
Goodness of fit on *F*^2^	0.819	1.059	1.196
*R*_1_, *ωR*_2_ [*I* > 2*σ*(I)] ^a^	0.0372, 0.1125	0.0324, 0.0854	0.0424, 0.1226
*R*_1_, *ωR*_2_ (all data)	0.0582, 0.1285	0.0408, 0.0909	0.0463, 0.1282

^a^ *R*_1_ = *∑*||*F*_0_| − *F*_c_||*∕*∑|*F*_0_|, *ωR*_2_ = [*∑*($F_{2}^{0}$ − $F_{2}^{c}$)/∑*ω*(*F*_0_)^2^]^1/2^, *ω* = [$\sigma_{c}^{2}$ ($F_{c}^{2}$) + (*χP*)^2^ + y*P*]^−1^, *P* = (*F*2 0 + 2$F_{c}^{2}$)/3.

**Table 2:** Selected Bond Lengths (Å) and Bond Angles (deg) of Compounds **1**–**3** ^a^.

	Compound 1 (Fe^II^)	Compound 2 (Co^II^)	Compound 3 (Cu^II^)
M-N(1)	2.201(2)	2.146(16)	2.041(13)
M-N(2)	2.154(2)	2.114(15)	2.014(14)
M-O(1)	2.146(2)	2.126(16)	2.420(18)
O(1)#1-Fe(1)-N(2)	89.51(8)	89.51(6)	89.38(5)
O(1)#1-Fe(1)-N(1)#1	89.95(8)	78.05(6)	92.55(5)
N(2)-Fe(1)-N(1)#1	103.22(8)	101.95(6)	99.22(6)
O(1)#1-Fe(1)-N(1)	90.05(8)	89.73(6)	87.45(5)

^a^ Symmetry transformations used to generate equivalent atoms: #1: *x* − 1, *y*, *z*.

**Table 3:** Hydrogen bonds for imports **1**–**3** (Å and °).

D–H···A	d(D–H)	d(H···A)	d(D···A)	<(DHA)
Compound **1**				
C(4)-H(4)···O(1)#1	0.93	3.06	3.830(2)	142.2
O(1)-H(1W)···N(4)#2	0.82(3)	1.942	2.831(3)	173(2)
O(1)-H(2W)···N(5)#3	0.69(3)	2.139	2.757(3)	171(2)
Compound **2**				
C(5)-H(5)···O(1)#1	0.93	2.56	3.343(3)	142.2
O(1)-H(1W)···N(4)#2	0.79(3)	2.05(3)	2.840(2)	174(3)
O(1)-H(2W)···N(5)#3	0.79(3)	1.99(3)	2.781(2)	176(3)
Compound **3**				
C(3)-H(3)···O(1)#1	0.92	2.68	3.883(3)	145.5
O(1)-H(1W)···N(4)#2	0.75(2)	2.06(2)	2.906(2)	179(2)
O(1)-H(2W)···N(5)#3	0.78(2)	2.16(2)	2.835(2)	171(2)

Symmetry transformations used to generate equivalent atoms: #1: *x* − 1, *y*, *z*; #2: *x*, −*y* + 3/2, *z* + 1/2; #3: −*x* + 2, *y*−1/2, −*z* + 1/2.

## Data Availability

All data generated or analyzed during this study are included in this published article.

## Supplementary Materials

The following supporting information can be downloaded at: https://www.mdpi.com/article/10.3390/molecules28186633/s1. Table S1: Provenance and Mass Fraction Purity (wt%) of the Chemicals Used in the Study. Table S2: Experimental Molar Magnetic Susceptibility Data of Compounds **1**–**3** at Constant Pressure (*p* = 1.2 mPa) and under 1000 Oe Applied Field (*T* = 2 to 350 K). Table S3: Experimental Molar Heat Capacities (*C_p_*_,m_) of Compounds **1**–**3** at Constant Pressure (*p* = 1.2 mPa) and under Zero Applied Field (*T* = 1.9–300 K). Table S4: Summary of the Fits of the Heat Capacity of Compounds **1**–**3** within the Temperature Range (1.9 to 300) K. Table S5: Standard Molar Specific Heat Capacity, Entropy and Enthalpy of Compounds **1**–**3** as a Function of Temperature *T* at the Standard Pressure *p* = 0.1 MPa and under Zero Applied Field.

## Sample Availability

Samples of the compounds are available from the authors.
