# Supplementary material for: Magnetic Property, Heat Capacity and Crystal Structure of Mononuclear Compounds Based on Substitute Tetrazole Ligand
Source: Molecules. 2023 Sep 15;28(18):6633. doi: 10.3390/molecules28186633 (PMC10535496; doi:10.3390/molecules28186633)
Supplement: Supplementary file 1 [file molecules-28-06633-s001.zip › molecules-2589389-supplementary.pdf]

Supplementary information

Magnetic Property, Heat Capacity and Crystal Structure of Mononuclear Compounds Based on Substitute Tetrazole Ligand

Hui Zheng [1,*,†], Jipeng Luo [2,†], Xiaoqin Wang [1], Nan Yin [2], Beibei Zhang [1], Xuezhen Gao [1], Zongzheng Zhang [1], Quan Shi [2] and Junshen Liu [1,*]

[1] School of Chemistry and Materials Science, Ludong University, Yantai 264025, China; wang20212023@163.com (X.W.); zhbb0302@163.com (B.Z.); gaoxuez@163.com (X.G.); zhangzongzheng@m.ldu.edu.cn (Z.Z.)

[2] Thermochemistry Laboratory, Dalian Technology Innovation Center for Energy Materials Thermodynamics, Liaoning Province Key Laboratory of Thermochemistry for Energy and Materials, Dalian National Laboratory for Clean Energy, Dalian Institute of Chemical Physics, Chinese Academy of Sciences, Dalian 116023, China; luojp@dicp.ac.cn (J.L.); yin7310@dicp.ac.cn (N.Y.); shiquan@dicp.ac.cn (Q.S.)

* Correspondence: zhenghui@ldu.edu.cn (H.Z.), liujunshen@163.com (J.L.)

† These authors contributed equally to this work.

Table and Figure of Contents

Table S1. Provenance and Mass Fraction Purity (*wt*%) of the Chemicals Used in the Study.

Table S2. Experimental Molar Magnetic Susceptibility Data of Compounds **1-3** at Constant Pressure (p = 1.2 mPa) and under 1000 Oe Applied Field (T = 2 to 350 K).

Table S3. Experimental Molar Heat Capacities ($C_{p,\mathrm{m}}$) of Compounds **1-3** at Constant Pressure (p = 1.2 mPa) and under Zero Applied Field (T = 1.9-300 K).

Table S4. Summary of the Fits of the Heat Capacity of Compounds **1-3** within the Temperature Range (1.9 to 300) K.

Table S5. Standard Molar Specific Heat Capacity, Entropy and Enthalpy of Compounds **1-3** as a Function of Temperature T at the Standard Pressure p = 0.1 MPa and under Zero Applied Field.

Table S1. Provenance and Mass Fraction Purity (*wt*%) of the Chemicals Used in the Study.

Formula	Source	State	*wt*%
Methanol	Beijing Chemical works, China	Liquid	99.0$^{\alpha}$
$Fe(ClO_4)_2·6H_2O$	Tianjin Hengxing Chemical Preparation Co. Ltd, China	Crystalline	99.0$^{\alpha}$
$Co(OAc)_2·6H_2O$	Tianjin Hengxing Chemical Preparation Co. Ltd, China	Crystalline	99.0$^{\alpha}$
$CuCl_2·2H_2O$	Tianjin Hengxing Chemical Preparation Co. Ltd, China	Crystalline	99.0$^{\alpha}$
2-(1H-tetrazol-5-yl)pyridine	Tianjin Hengxing Chemical Preparation Co. Ltd, China	Crystalline	99.0$^{\alpha}$
[M(2-(1H-tetrazol-5-yl)pyridine)$_2$ (H_2O)$_2$] (M = Fe^{II}/Co^{II}/Cu^{II})	Synthesized	Single crystal	98.9$^{\beta}$

$^{\alpha}$ Stated by the supplier; $^{\beta}$ Evaluated by the measured contents of C, H, and N.

The results are as follows, anal. calcd. (%) for C12H12N10O2Fe: C 37.48, H 3.12, N 36.44; experimental value: C 37.42, H 3.15, N 36.46. for C12H12N10O2Co: C 37.18, H 3.10, N 36.15; experimental value: C 37.21, H 3.11, N 36.13; for C12H12N10O2Cu: C 36.75, H 3.06, N 35.73; experimental value: C 36.70, H 3.12, N 35.69. It can be seen that the measured elemental analysis results are inconsistent with the calculated results. It is well known that the uncertainty of elemental analysis depends largely on the preparation of the sample used for the measurement, the homogeneity of the sample, the integrity of the combustion (reaction time), the selection of integration parameters, and the performance of the instrument. As for our element analysis measurement, the samples measured are not a single crystal but several crystals, and consequently the factors affecting the measurement uncertainties could be, (i) there may exist extra H2O on the crystal surface, (ii) the existence of lattice water or other free anions containing oxygen atoms, (iii) the instability of the crystal, and so on. In other words, due to the extensive presence of external C, N, and H elements, the element analysis experimental results may be inconsistent with the calculated results.

Table S2. Experimental Molar Magnetic Susceptibility Data of Compounds **1-3** at Constant Pressure (p = 1.2 mPa) and under 1000 Oe Applied Field (T = 2 to 350 K). $^{\delta}$

T K	χ_M cm^3 mol^{-1}	T K	χ_M cm^3 mol^{-1}	T K	χ_M cm^3 mol^{-1}	T K	χ_M cm^3 mol^{-1}
**Compound 1**							
2.00	1.269	90.03	0.044	180.03	0.025	266.06	0.018
4.02	0.781	92.77	0.043	182.66	0.024	268.07	0.018
6.01	0.553	94.01	0.042	184.02	0.024	270.11	0.018
8.08	0.427	96.08	0.042	186.01	0.024	272.87	0.018
10.06	0.349	98.01	0.041	188.09	0.024	274.00	0.018
14.59	0.249	100.00	0.040	190.03	0.024	276.01	0.018
16.00	0.226	104.01	0.039	193.90	0.023	278.07	0.018
18.07	0.200	106.02	0.038	194.01	0.023	280.05	0.018
20.13	0.180	108.02	0.038	196.02	0.023	284.09	0.018
22.03	0.165	110.07	0.037	198.07	0.023	286.12	0.018
25.44	0.144	112.04	0.036	200.07	0.023	288.02	0.017
26.03	0.140	115.20	0.036	202.07	0.023	290.06	0.017
28.09	0.130	116.04	0.035	205.14	0.022	292.06	0.017
30.01	0.122	118.07	0.035	206.07	0.022	295.43	0.017
32.07	0.115	120.03	0.034	208.05	0.022	296.10	0.017
34.20	0.108	122.03	0.034	210.04	0.022	298.03	0.017
36.65	0.101	126.46	0.033	212.07	0.022	300.01	0.017
38.03	0.097	128.04	0.033	214.03	0.022	302.09	0.017
40.08	0.093	130.01	0.032	216.45	0.021	304.07	0.017

42.07	0.088	132.06	0.032	218.02	0.021	306.79	0.017
44.08	0.085	134.03	0.031	220.08	0.021	308.07	0.017
47.81	0.078	137.66	0.031	222.05	0.021	310.03	0.017
48.08	0.078	138.06	0.031	224.03	0.021	312.03	0.016
50.05	0.075	140.05	0.030	227.73	0.021	314.00	0.016
52.15	0.072	142.01	0.030	228.01	0.021	318.04	0.016
54.06	0.070	144.02	0.030	230.03	0.020	320.08	0.016
56.12	0.068	146.03	0.029	232.11	0.020	322.06	0.016
58.98	0.064	149.08	0.029	234.07	0.020	324.05	0.016
60.11	0.063	150.08	0.029	236.05	0.020	326.03	0.016
62.07	0.062	152.04	0.028	238.92	0.020	329.39	0.016
64.04	0.060	154.05	0.028	240.00	0.020	330.06	0.016
66.05	0.058	156.01	0.028	242.03	0.020	332.08	0.016
70.27	0.055	160.15	0.027	244.04	0.020	334.06	0.016
72.07	0.054	162.03	0.027	246.07	0.020	336.03	0.016
74.00	0.052	164.09	0.027	250.22	0.019	338.03	0.016
76.06	0.051	166.05	0.026	252.02	0.019	340.76	0.016
78.01	0.050	168.01	0.026	254.00	0.019	342.05	0.016
81.55	0.048	171.40	0.026	256.06	0.019	344.03	0.016
82.01	0.048	172.03	0.026	258.05	0.019	346.02	0.015
84.08	0.047	174.05	0.025	261.54	0.019	348.01	0.015
86.00	0.046	176.05	0.025	262.06	0.019	349.95	0.015
88.01	0.045	178.05	0.025	264.07	0.019		
			**Compound 2**				
2.00	0.705	88.01	0.031	176.05	0.0154	264.01	0.0102
2.00	0.704	90.01	0.030	178.04	0.0151	266.07	0.0102
4.01	0.396	92.92	0.029	180.10	0.0150	268.07	0.0101
6.04	0.277	94.04	0.029	183.13	0.0147	270.02	0.0100
8.05	0.217	96.03	0.028	184.00	0.0147	273.14	0.0099
10.05	0.179	98.02	0.028	186.06	0.0145	274.05	0.0099
14.04	0.139	100.03	0.027	188.05	0.0144	276.06	0.0098
16.06	0.124	104.14	0.026	190.03	0.0142	278.08	0.0097
18.00	0.113	106.01	0.025	194.33	0.0139	280.04	0.0097
20.01	0.104	108.12	0.025	196.10	0.0138	284.40	0.0095
22.07	0.097	110.07	0.025	198.02	0.0136	286.03	0.0095
25.44	0.088	112.03	0.024	200.07	0.0135	288.07	0.0094
26.01	0.087	115.46	0.023	202.03	0.0134	290.06	0.0093
28.07	0.082	116.02	0.023	205.59	0.0131	292.08	0.0093
30.07	0.077	118.05	0.023	206.07	0.0131	295.70	0.0091
32.06	0.074	120.05	0.022	208.09	0.0130	296.02	0.0092
34.04	0.070	122.04	0.022	210.03	0.0129	298.05	0.0091
36.76	0.066	124.05	0.022	212.01	0.0128	300.05	0.0090
38.03	0.064	126.82	0.021	214.03	0.0126	302.01	0.0090
40.08	0.062	128.02	0.021	216.94	0.0124	304.04	0.0089
42.09	0.059	130.02	0.021	218.07	0.0124	307.07	0.0088
44.06	0.057	132.03	0.020	220.03	0.0123	308.05	0.0088
47.97	0.053	134.11	0.020	222.03	0.0121	310.08	0.0087
48.00	0.053	138.10	0.020	224.04	0.0120	312.06	0.0087
50.08	0.051	140.04	0.019	228.36	0.0118	314.07	0.0087
52.05	0.050	142.02	0.019	230.09	0.0117	318.43	0.0085
54.02	0.048	144.04	0.019	232.08	0.0116	320.04	0.0085
56.04	0.046	146.08	0.019	234.08	0.0116	322.02	0.0085
59.12	0.044	149.38	0.018	236.04	0.0115	324.03	0.0084
60.04	0.044	150.08	0.018	239.42	0.0113	326.01	0.0084
62.09	0.042	152.08	0.018	240.02	0.0113	329.72	0.0083
64.04	0.041	154.05	0.017	242.05	0.0112	330.05	0.0082
66.12	0.040	156.00	0.017	244.00	0.0111	332.01	0.0082

70.30	0.038	158.06	0.017	246.04	0.0110	334.07	0.0082
72.00	0.037	160.57	0.017	248.01	0.0109	336.04	0.0081
74.03	0.036	162.02	0.017	250.66	0.0108	338.08	0.0081
76.13	0.035	164.08	0.016	252.08	0.0107	341.10	0.0081
78.03	0.034	166.06	0.016	254.07	0.0106	342.05	0.0080
81.79	0.033	168.05	0.016	256.03	0.0105	344.03	0.0080
82.11	0.033	171.83	0.016	258.08	0.0105	346.01	0.0079
			**Compound 3**				
2.00	0.215	88.04	0.006	178.08	0.0040	268.05	0.0031
2.00	0.214	90.08	0.006	180.06	0.0039	270.07	0.0031
4.01	0.106	92.88	0.006	182.93	0.0039	273.15	0.0031
6.08	0.070	94.10	0.006	184.05	0.0039	274.02	0.0031
8.00	0.053	96.07	0.006	186.03	0.0039	276.06	0.0031
10.03	0.043	98.01	0.006	188.03	0.0038	278.04	0.0031
14.22	0.031	100.07	0.006	190.00	0.0038	280.02	0.0031
16.05	0.027	104.11	0.006	194.19	0.0038	282.02	0.0030
18.00	0.025	106.02	0.006	196.10	0.0037	284.45	0.0030
20.11	0.022	108.07	0.005	198.08	0.0037	286.03	0.0030
22.05	0.020	110.05	0.005	200.02	0.0037	288.06	0.0030
25.65	0.018	112.02	0.005	202.01	0.0037	290.04	0.0030
26.06	0.017	115.44	0.005	205.54	0.0036	292.06	0.0030
28.03	0.016	116.01	0.005	206.06	0.0036	295.76	0.0029
30.04	0.015	118.06	0.005	208.00	0.0036	296.05	0.0029
32.06	0.014	120.06	0.005	210.03	0.0036	298.01	0.0029
34.01	0.014	122.05	0.005	212.04	0.0036	300.08	0.0029
36.82	0.013	124.08	0.005	214.02	0.0036	302.09	0.0029
38.00	0.012	126.74	0.005	216.78	0.0035	304.07	0.0029
40.12	0.012	128.04	0.005	218.01	0.0035	307.04	0.0029
42.05	0.011	130.03	0.005	220.11	0.0035	308.06	0.0029
44.07	0.011	132.03	0.005	222.05	0.0035	310.03	0.0028
47.93	0.010	134.04	0.005	224.08	0.0034	312.03	0.0028
48.10	0.010	137.91	0.005	228.13	0.0034	314.01	0.0028
50.03	0.010	138.05	0.005	230.00	0.0034	318.31	0.0028
52.08	0.010	140.07	0.005	232.03	0.0034	320.08	0.0028
54.06	0.009	142.09	0.005	234.04	0.0034	322.08	0.0028
56.04	0.009	144.02	0.005	236.06	0.0034	324.06	0.0028
59.20	0.009	146.02	0.004	239.34	0.0034	326.09	0.0027
60.03	0.009	149.14	0.004	240.05	0.0033	329.67	0.0027
62.02	0.008	150.01	0.004	242.08	0.0033	330.04	0.0028
64.00	0.008	152.08	0.004	244.01	0.0033	332.06	0.0027
66.00	0.008	154.05	0.004	246.09	0.0033	334.01	0.0028
68.04	0.008	156.03	0.004	248.08	0.0033	336.12	0.0027
70.38	0.008	160.37	0.004	250.65	0.0033	338.02	0.0027
72.05	0.007	162.05	0.004	252.03	0.0033	340.98	0.0027
74.01	0.007	164.04	0.004	254.01	0.0032	342.02	0.0027
76.04	0.007	166.09	0.004	256.04	0.0032	344.05	0.0027
78.07	0.007	168.02	0.004	258.06	0.0032	346.02	0.0027
81.79	0.007	171.64	0.004	261.91	0.0032	348.00	0.0027
82.09	0.007	172.01	0.004	262.07	0.0032	349.96	0.0027
84.01	0.007	174.04	0.004	264.07	0.0031		
86.01	0.006	176.03	0.004	266.06	0.0031		

$^{\delta}$ The estimated standard uncertainties in the pressure p, temperature T, and magnetic field H are $u(p) = 0.10$ mPa, $u(T) = 0.01$ K ($2 < T$/K < 20), $u(T) = 0.02$ K ($20 < T$/K < 100), $u(T) = 0.05$ K ($100 < T$/K < 300), and $u(H) = 0.5$ Oe. The estimated standard uncertainties of the magnetic measurements are $u(\chi_M) = 0.05 \cdot \chi_M$ ($1.9 < T$/K < 350).

Table S3. Experimental Molar Heat Capacities ($C_{p,\mathrm{m}}$) of Compounds **1-3** at Constant Pressure (p = 1.2 mPa) and under Zero Applied Field (T = 1.9 to 300 K). $^{\zeta}$

T K	$C_{p,m}$ J·mol^{-1}·K^{-1}	T K	$C_{p,m}$ J·mol^{-1}·K^{-1}	T K	$C_{p,m}$ J·mol^{-1}·K^{-1}	T K	$C_{p,m}$ J·mol^{-1}·K^{-1}
			**Compound 1**				
1.942	0.120	9.165	8.985	43.867	135.024	171.692	494.150
2.141	0.152	10.175	11.685	48.674	153.844	181.793	517.730
2.375	0.197	11.295	14.892	54.033	175.672	191.888	540.765
2.632	0.259	12.538	18.766	59.975	198.229	201.995	560.633
2.918	0.340	13.920	23.332	66.573	222.574	212.136	580.143
3.238	0.452	15.450	28.495	73.909	243.654	222.215	605.679
3.592	0.606	17.156	34.276	81.999	273.722	232.318	629.634
3.986	0.827	19.040	40.853	91.019	300.414	242.387	658.301
4.409	1.095	21.134	48.411	101.027	328.540	252.537	682.872
4.896	1.512	23.457	56.554	111.109	357.155	262.680	707.246
5.443	2.031	26.040	65.962	121.230	382.312	272.766	731.691
6.035	2.763	28.902	76.652	131.292	406.523	282.786	755.605
6.702	3.763	32.076	88.838	141.426	428.106	292.936	779.187
7.440	5.095	35.609	101.717	151.531	448.578	302.992	805.447
8.257	6.807	39.526	117.806	161.632	472.861		
			**Compound 2**				
1.944	0.264	9.171	7.884	43.896	135.940	171.737	487.007
2.145	0.262	10.180	10.356	48.706	153.334	181.849	509.555
2.379	0.273	11.299	13.420	54.065	173.470	191.938	531.472
2.636	0.298	12.543	17.066	60.006	194.591	202.052	555.690
2.922	0.345	13.925	21.466	66.616	216.113	212.154	573.988
3.230	0.414	15.456	26.610	73.931	239.073	222.240	596.246
3.586	0.529	17.160	32.558	82.034	265.477	232.372	621.355
3.983	0.694	19.046	39.519	91.045	295.168	242.494	643.356
4.416	0.928	21.142	47.369	101.061	321.790	252.561	666.018
4.898	1.252	23.464	56.249	111.198	347.505	262.655	689.734
5.435	1.692	26.048	66.352	121.237	371.565	272.799	715.373
6.034	2.303	28.911	77.574	131.370	394.835	282.819	739.033
6.698	3.189	32.082	90.423	141.457	422.179	292.952	760.649
7.441	4.382	35.615	103.712	151.536	441.213	303.071	792.700
8.262	5.929	39.547	119.295	161.640	463.062		
			**Compound 3**				
1.921	7.888	3.642	7.973	5.051	8.446	12.537	20.554
1.925	7.851	3.742	8.027	5.051	8.445	13.916	24.604
2.028	7.812	3.743	8.027	5.052	8.408	15.446	29.480
2.128	7.802	3.743	8.031	5.150	8.494	17.148	35.029
2.129	7.769	3.843	8.069	5.152	8.469	19.032	41.499
2.140	7.757	3.845	8.049	5.152	8.453	23.450	57.057
2.230	7.744	3.846	8.067	5.253	8.434	26.027	66.514
2.331	7.717	3.944	8.129	5.253	8.484	28.893	77.186
2.333	7.708	3.945	8.111	5.255	8.517	32.064	89.307
2.374	7.702	3.946	8.111	5.353	8.525	35.591	102.385
2.432	7.698	3.991	8.103	5.353	8.471	39.528	117.550

2.533	7.693	4.043	8.173	5.354	8.527	43.863	134.510
2.534	7.690	4.046	8.152	5.451	8.510	48.682	151.299
2.633	7.682	4.046	8.159	5.454	8.558	54.031	172.179
2.633	7.679	4.146	8.201	5.455	8.515	59.970	193.743
2.733	7.695	4.147	8.205	5.456	8.542	66.598	213.711
2.734	7.688	4.147	8.186	5.556	8.543	73.904	236.773
2.833	7.706	4.242	8.247	5.556	8.574	82.022	263.452
2.919	7.716	4.242	8.252	5.558	8.580	91.016	292.580
2.934	7.710	4.248	8.227	5.656	8.577	101.016	318.749
2.934	7.712	4.349	8.284	5.656	8.624	111.140	347.475
3.034	7.740	4.353	8.191	5.658	8.584	121.242	371.219
3.135	7.754	4.440	8.295	5.757	8.610	131.299	394.749
3.136	7.756	4.446	8.338	5.758	8.633	141.429	416.592
3.234	7.800	4.446	8.298	5.760	8.619	151.527	438.904
3.235	7.794	4.543	8.230	5.857	8.705	161.640	461.228
3.237	7.787	4.547	8.302	5.858	8.669	171.735	484.038
3.240	7.770	4.550	8.263	5.860	8.672	181.845	506.826
3.338	7.832	4.647	8.362	5.959	8.721	191.949	528.128
3.338	7.830	4.648	8.333	5.959	8.719	202.063	549.181
3.339	7.813	4.649	8.284	5.961	8.706	212.179	570.942
3.439	7.889	4.746	8.403	6.047	8.765	222.269	594.645
3.439	7.880	4.747	8.392	6.059	8.771	232.354	619.987
3.440	7.874	4.750	8.357	6.060	8.779	242.446	644.004
3.540	7.920	4.849	8.409	6.709	9.236	252.597	666.941
3.540	7.921	4.852	8.400	7.443	10.011	262.715	689.680
3.541	7.921	4.915	8.404	8.263	11.035	272.741	714.374
3.595	7.940	4.949	8.423	9.170	12.514	282.905	740.304
3.641	7.965	4.949	8.428	10.177	14.548	292.981	760.399
3.642	7.970	4.950	8.389	11.295	17.233	303.071	786.174

$^{\zeta}$ The estimated standard uncertainties in the pressure p and temperature T are $u(p)$ = 0.10 mPa, and $u(T)$ = 0.01 K (2 < T/K < 20), $u(T)$ = 0.02 K (20 < T/K < 100), and $u(T)$ = 0.05 K (100 < T/K < 300). The standard uncertainties of the heat capacity measurements are $u(C_{p,m}) = 0.03 \cdot C_{p,m}$ (1.9 < T/K < 20) and $u(C_{p,m}) = 0.01 \cdot C_{p,m}$ (20 < T/K < 300).

Table S4. Summary of the Fits of the Heat Capacity of Compounds **1-3** within the Temperature Range (1.9 to 300) K.

Parameters	**1**	**2**	**3**
	Low T fits		
γ / ($J \cdot mol^{-1} \cdot K^{-2}$)	0.018317	/	1.3445
B_3 / ($J \cdot mol^{-1} \cdot K^{-4}$)	0.011664	9.4494E-3	0.04921
B_5 / ($J \cdot mol^{-1} \cdot K^{-6}$)	-5.60465E-5	4.1214E-5	-2.4385E-3
B_7 / ($J \cdot mol^{-1} \cdot K^{-8}$)	/	-3.7954E-7	-1.0570E-5
n_{sch} / (mol)	0.014218	0.089566	1.5292
θ / (K)	10.529	2.3447	3.2684
B_{asw} / ($J \cdot mol^{-1} \cdot K^{-2}$)	/	/	3.6286
Δ / (K)	/	/	25.572
%RMS	0.4531	0.5266	0.2546
Range / (K)	1.9~7.16	1.9~7.88	1.9~6.24
	Middle T fits		

A_0 / (J·mol^{-1}·K^{-1})	8.7670	5.4209	17.679
A_1 / (J·mol^{-1}·K^{-2})	-4.2153	-2.6413	-4.0158
A_2 / (J·mol^{-1}·K^{-3})	0.7141	0.43335	0.51778
A_3 / (J·mol^{-1}·K^{-4})	-3.5439E-2	-0.01505	-0.018163
A_4 / (J·mol^{-1}·K^{-5})	1.0107E-3	2.8815E-4	3.4867E-4
A_5 / (J·mol^{-1}·K^{-6})	-1.6340E-5	-2.8628E-06	-3.4276E-6
A_6 / (J·mol^{-1}·K^{-7})	1.3936E-7	1.1385E-08	1.3358E-8
A_7 / (J·mol^{-1}·K^{-8})	-4.8970E-10	/	/
%RMS	0.1874	0.2391	0.3694
Range / (K)	7.16~61.60	7.88~55.70	6.24~54.20
	High T fits		
n_D/(mol)	12.089	13.047	11.300
Θ_D/(K)	194.49	207.85	186.73
$n_{E,1}$/(mol)	12.775	13.924	13.029
$\Theta_{E,1}$/(K)	446.67	521.00	442.98
$n_{E,2}$/(mol)	41.652	43.373	38.190
$\Theta_{E,2}$/(K)	1354.8	1482.1	1325.0
%RMS	0.3494	0.4721	0.2979
Range / (K)	61.60~300	55.70~300	54.20~300

Table S5. Standard Molar Specific Heat Capacity, Entropy and Enthalpy of Compounds **1-3** as a Function of Temperature T at the Standard Pressure p = 0.1 MPa and under Zero Applied Field. $^{\theta}$

T<br>K	$C^{o}_{p,\mathrm{m}}$<br>J·mol^{-1}·K^{-1}	$\Delta_0^T H^{o}_m$<br>J·mol^{-1}	$\Delta_0^T S^{o}_m$<br>J·mol^{-1}·K^{-1}	T<br>K	$C^{o}_{p,\mathrm{m}}$<br>J·mol^{-1}·K^{-1}	$\Delta_0^T H^{o}_m$<br>J·mol^{-1}	$\Delta_0^T S^{o}_m$<br>J·mol^{-1}·K^{-1}
			Compound **1**				
0	0.00	0.00	0.00	70	233.04	7267.75	171.54
1	0.03	0.01	0.02	75	249.72	8474.84	188.19
2	0.13	0.08	0.07	80	265.96	9764.21	204.83
3	0.37	0.32	0.16	85	281.76	11133.69	221.43
4	0.83	0.89	0.32	90	297.11	12581.06	237.97
5	1.59	2.07	0.58	95	311.99	14104.01	254.44
6	2.72	4.20	0.97	100	326.38	15700.15	270.81
7	4.27	7.66	1.50	110	353.69	19102.17	303.21
8	6.25	12.89	2.19	120	379.14	22767.81	335.09
9	8.58	20.27	3.06	130	403.00	26679.69	366.39
10	11.20	30.14	4.10	140	425.63	30823.66	397.09
11	14.05	42.75	5.30	150	447.46	35189.61	427.20
12	17.08	58.30	6.65	160	468.88	39771.52	456.77
13	20.25	76.95	8.14	170	490.23	44567.05	485.83
14	23.52	98.83	9.76	180	511.77	49576.84	514.46
15	26.88	124.02	11.49	190	533.68	54803.75	542.72
16	30.30	152.61	13.34	200	556.06	60252.06	570.66
17	33.77	184.64	15.28	210	578.96	65926.73	598.34
18	37.26	220.15	17.31	220	602.35	71832.86	625.81
19	40.79	259.18	19.42	230	626.19	77975.22	653.11
20	44.33	301.73	21.60	240	650.40	84357.92	680.27
25	62.26	568.05	33.41	250	674.89	90984.20	707.32

30	80.66	925.12	46.38	260	699.55	97856.30	734.27
35	99.74	1375.84	60.24	270	724.28	104975.42	761.13
40	119.46	1923.64	74.84	273.15	732.06	107269.16	769.58
45	139.51	2571.03	90.07	280	748.97	112341.73	787.92
50	159.49	3318.64	105.80	290	773.54	119954.42	814.63
55	179.14	4165.39	121.93	298.15	793.40	126339.81	836.35
60	198.37	5109.34	138.34	300	797.89	127811.75	841.27
65	215.88	6145.25	154.92				
			Compound **2**				
0	0.00	0.00	0.00	70	228.63	7212.36	169.88
1	0.34	0.16	0.22	75	244.24	8394.74	186.18
2	0.26	0.45	0.43	80	259.40	9654.00	202.43
3	0.36	0.74	0.55	85	274.19	10988.11	218.61
4	0.70	1.25	0.69	90	288.69	12395.44	234.69
5	1.32	2.24	0.91	95	302.91	13874.54	250.68
6	2.28	4.01	1.23	100	316.84	15424.03	266.57
7	3.64	6.93	1.68	110	343.83	18728.40	298.05
8	5.41	11.43	2.28	120	369.59	22296.52	329.08
9	7.50	17.85	3.03	130	394.11	26115.99	359.64
10	9.89	26.53	3.94	140	417.52	30174.99	389.71
11	12.54	37.73	5.01	150	440.02	34463.33	419.28
12	15.41	51.68	6.22	160	461.86	38973.17	448.38
13	18.46	68.61	7.57	170	483.32	43699.27	477.03
14	21.68	88.67	9.06	180	504.62	48638.99	505.26
15	25.03	112.01	10.67	190	526.00	53791.98	533.11
16	28.50	138.77	12.39	200	547.59	59159.68	560.64
17	32.06	169.05	14.23	210	569.51	64744.89	587.88
18	35.70	202.92	16.16	220	591.82	70551.21	614.89
19	39.41	240.47	18.19	230	614.53	76582.65	641.70
20	43.16	281.75	20.31	240	637.64	82843.19	668.34
25	62.36	545.34	31.99	250	661.09	89336.53	694.84
30	81.82	905.77	45.08	260	684.82	96065.86	721.23
35	101.28	1363.53	59.16	270	708.77	103033.68	747.53
40	120.69	1918.48	73.95	273.15	716.35	105278.24	755.79
45	139.99	2570.26	89.28	280	732.86	110241.75	773.74
50	158.94	3317.78	105.02	290	757.00	117691.03	799.88
55	177.20	4158.50	121.03	298.15	776.66	123940.70	821.13
60	195.29	5089.92	137.22	300	781.11	125381.64	825.95
65	212.39	6109.52	153.54				
			Compound **3**				
0	0.00	0.00	0.00	70	225.73	7229.29	189.81
1	6.39	2.27	3.44	75	241.73	8398.09	205.93
2	7.84	9.93	8.70	80	257.41	9646.07	222.04
3	7.73	17.64	11.83	85	272.74	10971.57	238.10
4	8.13	25.56	14.10	90	287.70	12372.82	254.12
5	8.43	33.85	15.95	95	302.25	13847.86	270.07

6	8.74	42.41	17.51	100	316.38	15394.61	285.93
7	9.49	51.48	18.91	110	343.29	18694.41	317.36
8	10.71	61.54	20.25	120	368.49	22254.65	348.32
9	12.33	73.03	21.60	130	392.20	26059.21	378.76
10	14.29	86.31	23.00	140	414.79	30094.95	408.66
11	16.55	101.71	24.47	150	436.62	34352.43	438.03
12	19.07	119.50	26.01	160	458.06	38825.98	466.89
13	21.81	139.93	27.65	170	479.41	43513.29	495.30
14	24.74	163.19	29.37	180	500.91	48414.71	523.31
15	27.83	189.47	31.18	190	522.71	53532.50	550.98
16	31.04	218.89	33.08	200	544.88	58870.09	578.35
17	34.37	251.59	35.06	210	567.45	64431.42	605.48
18	37.78	287.66	37.12	220	590.41	70220.43	632.40
19	41.27	327.18	39.26	230	613.68	76240.64	659.16
20	44.81	370.21	41.46	240	637.20	82494.88	685.78
25	63.01	639.51	53.41	250	660.87	88985.13	712.27
30	81.55	1000.86	66.53	260	684.59	95712.43	738.65
35	100.22	1455.21	80.50	270	708.28	102676.87	764.93
40	119.08	2003.37	95.11	273.15	715.72	104919.66	773.19
45	138.13	2646.36	110.24	280	731.83	109877.56	791.11
50	157.15	3384.66	125.78	290	755.17	117312.79	817.20
55	175.15	4216.66	141.62	298.15	773.98	123544.22	838.39
60	192.57	5136.28	157.62	300	778.22	124980.00	843.19
65	209.38	6141.36	173.70				

$^{\theta}$All calculated thermodynamic values have estimated standard uncertainty of about 0.03X below 20 K and 0.01X above 20 K, where X represents the thermodynamic properties.
